# Editorial: Unveiling the potential of CTCs in drug resistance mechanisms and personalized medicine

**DOI:** 10.3389/fonc.2024.1519816

**Published:** 2024-11-26

**Authors:** Chiara Nicolazzo, Elisa Frullanti, Paola Gazzaniga, Maria Palmieri

**Affiliations:** ^1^ Cancer Liquid Biopsy Unit, Department of Molecular Medicine, Sapienza University of Rome, Rome, Italy; ^2^ Cancer Genomics & Systems Biology Lab, Department of Medical Biotechnologies, University of Siena, Siena, Italy; ^3^ Med Biotech Hub and Competence Centre, Department of Medical Biotechnologies, University of Siena, Siena, Italy

**Keywords:** cancer, liquid biopsy, treatment resistance, circulating biomarkers, cancer progression

## Introduction

Cancer remains a leading cause of mortality worldwide and the field of oncology has seen significant advancements with the discovery and study of Circulating Tumor Cells (CTCs). First described more than 150 years ago (1), the CTC are released into the bloodstream from primary and metastatic tumors, potentially forming new tumors in distant organs. CTCs are critical components of liquid biopsies, offering a noninvasive and dynamic window into tumor biology. They provide invaluable insights into cancer dissemination, disease progression, and response to treatment, reflecting tumors spatial and temporal heterogeneity (2). A high CTC count has been associated with poor prognosis in several cancers and at various disease stages, especially in breast, lung, and prostate cancer and for this reason, to date, their use is mainly for prognostic purposes (3).

However, the potential of CTCs should not remain confined only to prognostic study but be extended also to clinical practice. Therefore, it is of pivotal importance to explore the clinical relevance of CTCs beyond their prognostic value, studying the emerging methods application for CTCs to use the information deriving from CTCs in the treatment of patients through precision therapy. The information provided by CTCs about genetic mutations, gene expression patterns, and biomarkers can be used to identify specific molecular targets for understanding drug resistance mechanisms and drug development, leading to personalized treatment options that can prevent metastasis and improve patient outcomes.

This Research Topic focused on the recognition methods of CTCs based on deep learning and emerging tools to investigate the genome, transcriptome, proteome, and secretome of CTCs as biomarkers.

## Research progress for circulating tumor cells

In this Research Topic, two original research and three reviews have been published regarding new scientific advances in the CTCs. In particular, Chu et al. study the potential of peptide magnetic nanoparticles (Pep@MNP) in capturing CTCs in pancreatic ductal adenocarcinoma. CTCs and CXCR4 expression were detected to explore their clinical significance. Indeed, the CXCR4+ CTCs were found to be associated with early recurrence and metastasis of PDAC. Therefore, CTCs identified by Pep@MNP detection system could be used as diagnostic and prognostic biomarkers of PDAC patients.

Ntzifa et al. focus their research on the clinical outcomes of NSCLC patients who eventually develop resistance to osimertinib. They combined plasma-cfDNA and CTC analysis to find possible resistance mechanisms and druggable targets. They concluded on the efficiency of comprehensive liquid biopsy analysis to represent the heterogeneous molecular landscape and to provide prominent information on subsequent treatments for NSCLC patients at disease progression since druggable molecular alterations were detected in CTCs.

Particular attention has been paid to summarizing the progress made in the field of liquid biopsy by Wang et al. In their review article, they focus on the three main branches of liquid biopsy the CTC, the circulating tumor DNA (ctDNA), and the extracellular vehicles (EVs) even though already been employed in several clinical trials, the clinical utility of these three is still being studied, with promising initial findings. The review gives an overview of the emerging technologies for the isolation, characterization, and content detection of CTC, ctDNA, and EVs.

Also, Zhang et al. addressed the progress of liquid biopsy but from the point of view of the cell membrane biomimetic nanoparticles for circulating tumor cells. These biomimetic nanoparticles covered with cell membranes using functional, targeted, and biocompatible coating technology can target specific cells, stay in circulation for longer, and avoid immune responses, which makes them much better at capturing CTCs. The review article examines the current opportunities and difficulties associated with using cell membrane–coated nanoparticles as a capture technique for CTCs in addition to its growing clinical possibilities practices.

An interesting review article on advances in circulating tumor cells for early detection, prognosis and metastasis reduction in lung cancer was addressed by Wang et al. They have enumerated both *in vivo* and *ex vivo* techniques for CTC separation and enrichment, examined the advantages and limitations of these methods and also discussed the detection of CTCs in other bodily fluids. Furthermore, they evaluated the CTCs in conjunction with other biomarkers, for their utility in the early detection and prognostic assessment of patients with lung cancer.

Finally, Smilkou et al. compared the efficacy of the ultrasensitive real-time PCR assay with droplet digital PCR (ddPCR) for the detection of the three most common hotspot mutations of PIK3CA, in exons 9 and 20, that are of clinical importance in several cancers. They evaluated accuracy and sensitivity of this two method in plasma cell-free DNA and paired CTC-derived gDNAs. They demonstrated that the low-cost and widely used ultrasensitive real-time PCR assay provides accurate and specific detection of PIK3CA hotspot mutations in liquid biopsy samples giving similar results to ddPCR. However, PIK3CA mutations detected in CTC-derived gDNA and paired plasma-cfDNA were not identical, revealing that CTC and cfDNA give complementary picture of cancer development and status over time.

## Conclusions

In conclusion, this Research Topic highlights the importance of exploring the clinical relevance of CTCs beyond their prognostic value. The key findings discussed herein demonstrate that the studies on liquid biopsy for its use in tumors are in constant and unstoppable growth, and the strong interest in this field of research, supported by these scientific articles, gives us hope that the data we will collect with future research on the clinical applicability of CTCs will soon be used in clinical practice.
